# Asymmetric Rectified Electric Fields for Symmetric Electrolytes

**DOI:** 10.1021/acs.langmuir.4c01516

**Published:** 2024-06-25

**Authors:** A. Barnaveli, R. van Roij

**Affiliations:** Institute for Theoretical Physics, Center for Extreme Matter and Emergent Phenomena, Utrecht University, Princetonplein 5, Utrecht 3584 CC, The Netherlands

## Abstract

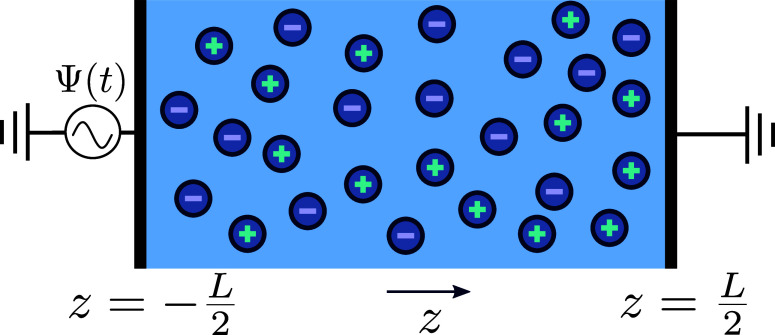

In this paper, building upon the numerical discovery of asymmetric rectified electric fields (AREFs), we explore the generation of AREF by applying a sawtooth-like voltage to 1:1 electrolytes with equal diffusion coefficients confined between two planar blocking electrodes. This differs from an earlier approach based on a sinusoidal AC voltage applied to 1:1 electrolytes with unequal diffusion coefficients. By numerically solving the full Poisson–Nernst–Planck equations, we demonstrate that AREF can be generated by a slow rise and a fast drop of the potential (or vice versa), even for electrolytes with equal diffusion coefficients of the cations and anions. We employ an analytically constructed equivalent electric circuit to explain the underlying physical mechanism. Importantly, we find that the strength of AREF can be effectively tuned from zero to its maximal value by only manipulating the time dependence of the driving voltage, eliminating the necessity to modify the electrolyte composition between experiments. This provides valuable insights to control the manipulation of AREF, which facilitates enhanced applications in diverse electrochemical systems.

## 1 Introduction

Studying the behavior of an aqueous electrolyte subjected to an externally applied oscillating electric field often involves the use of alternating current (AC) voltages. For instance, an AC voltage is commonly used in areas such as induced charge electrokinetics,^1−5^ particle assembly in electrolytes,^6−13^ AC electroosmosis,^14−20^ cyclic voltammetry,^21−26^ batteries,^27−34^ sensing,^15,17,35^ and impedance spectroscopy.^28−34,36,37^ One of the main reasons for choosing AC electric fields over DC fields in various applications is to avoid any net current or net charge in the system, since the field has a zero mean over one cycle.

A basic geometry that can capture many of the essential physical effects of an AC field is a globally neutral 1:1 electrolyte of point-like ions confined between two blocking electrodes and subjected to a harmonic AC voltage. If the frequency of the AC voltage is relatively low or zero (as in equilibrium), then a so-called electric double layer (EDL), consisting of the surface charges of the solid and a diffuse ionic cloud with opposite charge, will form at the interface between a charged solid (electrode, colloid, etc.) and an electrolyte. The EDL harbors a surplus of counterions and a reduced concentration of co-ions compared to the bulk, thereby screening the electric field of the electrode. The typical thickness of a fully formed EDL is equal to the Debye length λ_D_, which is about 10 nm for water with 1 mM salt concentration at room temperature. One of the interesting recent findings in such a (vertical) system with horizontal electrodes concerned colloids floating in the gravitational field. Here, charged colloids suspended in an aqueous electrolyte were confined between two horizontal blocking electrodes that were driven by a harmonic AC potential. Contrary to intuition, rather than sedimenting in the gravitational field, the colloidal particles were observed to float against the gravitational pull.^38,39^ This led to a theoretical investigation to elucidate the source of the force that allows the colloids to withstand the gravitational field. In ref (40), it was shown that period-averaged electrode charge is not necessarily zero in the case of cations (+) and anions (−) with unequal diffusion coefficients, *D*_+_ ≠ *D*_–_. The resulting period-averaged induced electric field is therefore also nonzero and stretches from the electrodes well into the bulk of the electrolyte. It was termed the asymmetric rectified electric field (AREF). The electric force generated by AREF was proposed as a mechanism that would enable the colloids to counteract the gravitational pull. It is noteworthy that a recent study has proposed alternative mechanisms for colloidal floating, including dielectrophoresis (DEP) or electrohydrodynamical mechanisms.^41^ Interestingly, the predominant contribution of each mechanism to the floating height of colloids remains a subject of investigation.

**Figure 1:**
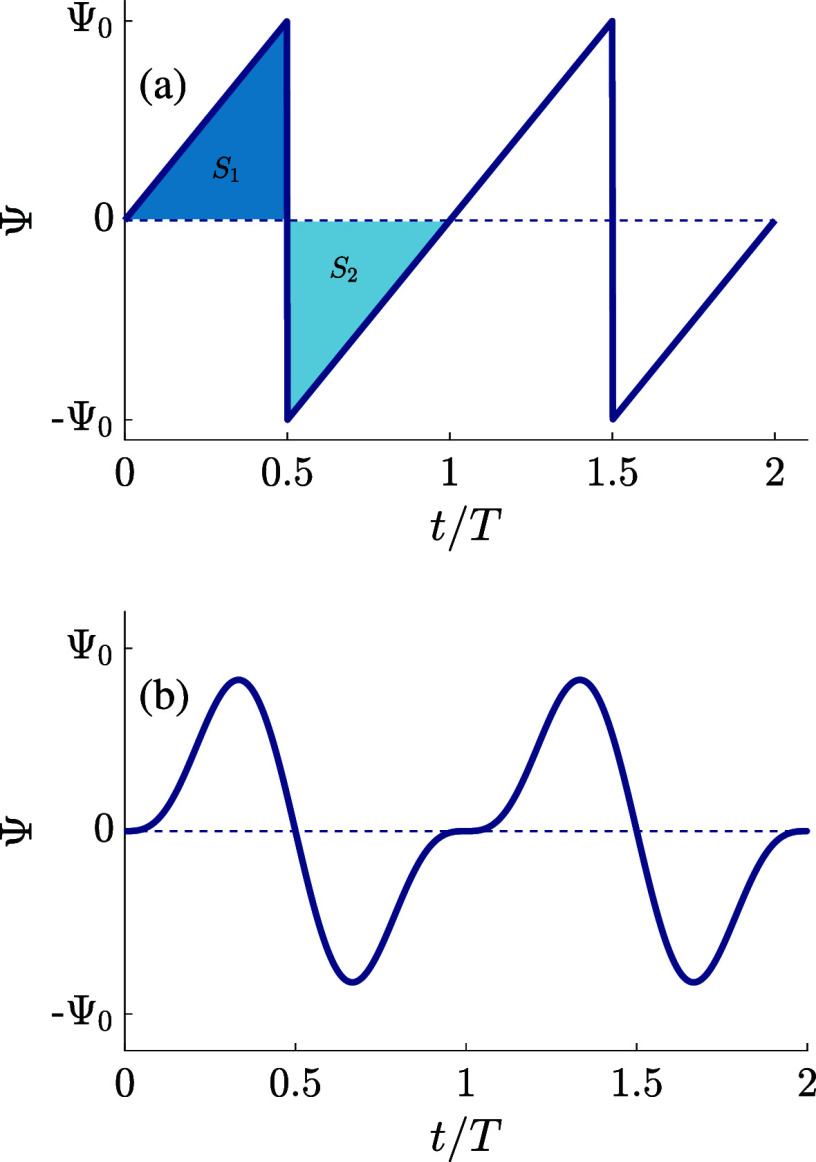
Two periods of the (a) full sawtooth voltage Ψ(*t*) based on eq 1 and (b) two-term sawtooth voltage based on eq 2. Both voltages have a period *T*, feature an asymmetry between (slow) rising and (fast) lowering voltages, and average out to zero during a period. The two-term sawtooth avoids sharp transitions, rendering itself more convenient for numerical calculations.

This paper focuses on AREF. The authors of the original study extensively explored AREF from sinusoidal voltages by examining its space dependence on various system parameters in ref (42), numerically solving the governing system of nonlinear differential equations in ref (43) and investigating the application of AREF in reversing the flow of electroosmosis in ref (44). Other studies have analyzed AREF analytically as well^45^ and considered multicomponent electrolytes.^46^ Nevertheless, several aspects of the underlying physical mechanism of AREF remained unclear. In our recent publication,^47^ we employ equivalent electric circuits to devise a simplified toy model that qualitatively reproduces the parameter dependencies of AREF, shedding light on the underlying physical mechanism. It was explained how the asymmetry of ion diffusion coefficients in the electrolyte can create AREF. However, the scope of manipulating AREF is constrained by the rather limited range of ion diffusion coefficients and their disparities. Furthermore, experimental studies on AREFs necessitate altering electrolytes for each new experiment, demanding a significant investment of time and effort. To address these challenges, we opted to study one and the same electrolyte, for simplicity, a symmetric 1:1 electrolyte with equal ion diffusion coefficients *D* ≡ *D*_+_ = *D*_–_, and instead study the possibility of introducing the necessary asymmetry for AREF generation through the functional form of the driving potential. A convenient form that is both asymmetric and periodic, yet averages to zero over time, is the so-called “sawtooth” potential
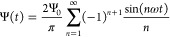
1where Ψ_0_ > 0 is the amplitude and *T* = 2π/ω is the period of the driving voltage Ψ(*t*). In Figure 1a, where we plot two periods of Ψ(*t*) given by eq 1 as a function of the dimensionless time *t*/*T*, we see that the sawtooth function rises steadily toward its maximum Ψ_0_ and then drops “instantaneously” to its minimum – Ψ_0_. This slow rise and fast drop breaks the symmetry of the charging and discharging processes at the electrodes, as we will see. At the same time, the (absolute) areas *S*_1_ and *S*_2_ under the curve are equal, *S*_1_ = *S*_2_, resulting in a period-averaged applied potential equal to zero, i.e., there is no direct bias of the voltage.

While the full sawtooth function is indeed a very convenient candidate for the time dependence of the driving voltage, it is less attractive for the numerical study that we undertake in this work, not only because of the large number of required harmonic “modes” in eq 1 but also because of the discontinuity of the full potential. It turns out that the essence of the creation mechanism of AREF can be studied in full detail by avoiding the sharpest feature of the full potential and keeping only the first two terms in the sawtooth series of eq 1. Thus, henceforth, the driving voltage of interest is given by
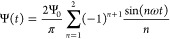
2which is plotted in Figure 1b. One checks that the role of the second harmonic term is to break the symmetry between rising and lowering voltages. All numerical results in this paper will be based on this “two-term” sawtooth function, that captures the key physics even though its actual amplitude is only ∼0.9Ψ_0_. However, for convenience and clarity, we will refer to the full sawtooth function when explaining and discussing the AREF mechanism. It should be noted here that the potential of eq 2 is similar to the one considered in ref (48), where, among other findings, the possibility of creating the AREF using “nonantiperiodic” potentials was demonstrated. In the current paper, we concentrate on the AREF induced by the sawtooth style potential to study its parameter dependence and explain the physical mechanism behind it, allowing us to understand its observed unexpected behavior in certain parameter regimes discussed in the “Results and Discussion” section below.

**Figure 2:**
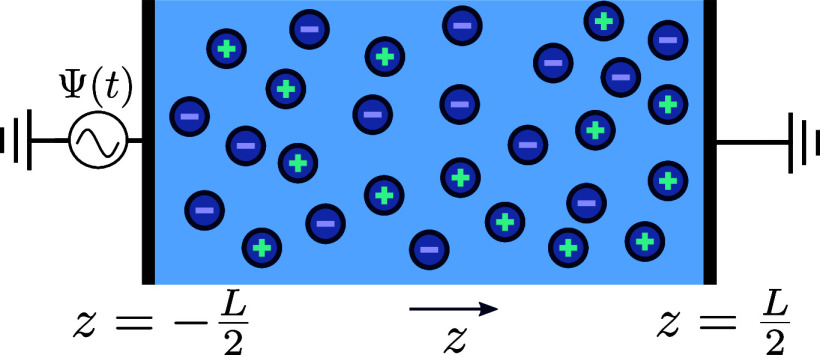
Schematic illustration of the aqueous 1:1 electrolyte under consideration, comprising a continuous solvent and two ionic species, enclosed between two parallel blocking electrodes with a separation distance *L*. The ions in the electrolyte are driven by the time-dependent electric sawtooth potential Ψ(*t*) of eq 2 applied to the electrode at , while the opposite electrode at  remains grounded.

The system of interest, schematically illustrated in Figure 2, is essentially the same electrolytic cell as the one considered in our previous paper,^47^ therefore its description and the notation we use will follow ref (47) very closely. The cell comprises a three-dimensional aqueous electrolyte with a relative dielectric constant ε at room temperature, confined between two parallel macroscopic planar electrodes separated by a distance *L*. We assume translational symmetry in the lateral directions. Apart from the continuum solvent, the electrolyte is composed of two types of monovalent point-like ions: cations (+) and anions (−) with valencies ±1 and equal diffusion coefficients *D*_±_ ≡ *D*. The total number of cations and anions is equal, ensuring overall electroneutrality in the system. The electrodes are blocking, preventing ions from leaving the electrolyte, and we exclude any chemical REDOX reactions. The system is subjected to the AC sawtooth voltage of eq 2 containing only two terms in the series, applied to the left electrode placed in the plane , whereas the right one, situated at , remains grounded. The imposed angular frequency is denoted by ω, and Ψ_0_ represents the amplitude of the applied voltage.

We study this system in terms of the Poisson–Nernst–Planck (PNP) equations. The ionic fluxes, denoted as *J*_±_(*z*, *t*), comprise a diffusive component arising from ion concentration gradients and a conductive component resulting from the potential gradient. These aspects are collectively described by the Nernst–Planck equation given by
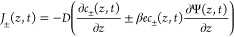
3where *c*_±_(*z*, *t*) represents the concentrations of cations (+) and anions (−) at the position *z* and time *t*, and Ψ(*z*, *t*) is the local electrostatic potential. Here, *e* is the elementary charge and β^–1^ is the product of the Boltzmann constant and the temperature. Equation 3 also assumes spatially constant diffusion coefficients. Given the absence of chemical reactions in the system, the concentrations and fluxes are connected through the continuity equation
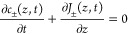
4

The local potential profile Ψ(*z*, *t*) is connected to the local charge density *e*(*c*_+_(*z*,*t*) – *c*_–_(*z*,*t*)) through the Poisson equation, which for  reads
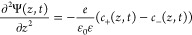
5where ε_0_ is the permittivity of vacuum and ε = 80 represents water as a structureless continuum.

The PNP eqs 3–5 form a closed set that fully describes the time-dependent profiles of the concentrations *c*_±_, the fluxes *J*_±_, and the potential Ψ. The explicit solution of the PNP equations requires boundary and initial conditions, for which we take
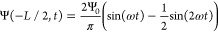
6
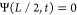
7
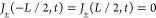
8
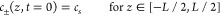
9Here, *c*_s_ represents the constant initial salt concentration, which is identical for both ionic species in the 1:1 electrolyte of interest here and thus satisfies global charge neutrality. As implied by eq 4 coupled with the boundary conditions specified in eq 8, the total number of anions and cations in the system is conserved such that
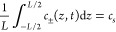
10is satisfied at all times *t* ≥ 0. For a given set of parameters Ψ_0_, ω, *D*, *c*_s_, and *L*, eqs 5–10 constitute the system of nonlinear coupled differential equations. We employ the finite-element solver of COMSOL to numerically solve these equations.

Convenient insights into relevant dimensionless system parameters can be obtained as follows. In the static (low-frequency) limit equilibrium holds, the applied potential Ψ(−*L*/2, *t*) = Ψ_0_ is a time-independent constant and *J*_±_(*z*, *t*) = 0. In the linear-screening regime with |β*e*Ψ_0_|≲ 1, the EDLs get fully developed at the two electrodes and the NP eq 3 can be integrated to obtain the Boltzmann distribution
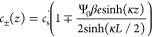
11with κ^–1^ being the characteristic Debye length of the equilibrium EDL given by
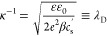
12

The concentration *c*_s_^′^ is an integration constant that is very close to *c*_s_ in the large *L*-limit of interest here, so throughout the paper, we set *c*_s_^′^ = *c*_s_ in the definition of λ_D_. In this limit, as we have shown before in ref (47), the characteristic time scale of EDL formation^49^ is written as the *RC* time
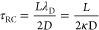
13

For future convenience, we also define the Debye time
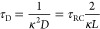
14during which the ions diffuse over a distance of the order of the Debye length.^49,50^

For the convenience of numerical investigation of AREF, we establish a standard parameter set that includes the (dimensionless) amplitude and frequency of the driving potential, denoted as β*e*Ψ_0_ = 3 and ωτ_RC_ = 1, respectively. The standard (dimensionless) system size is fixed at κ*L* = 50. We note that this standard parameter set is physically realistic, as it corresponds for an aqueous 1:1 electrolyte with a salt concentration *c*_s_ = 1 mM to a Debye length λ_D_ = 10 nm and hence a system length *L* = 500 nm, and with a typical diffusion coefficient *D* = 1.09 μm^2^/ms, we find τ_RC_ = 2.3 μs and hence a driving period *T* = 14.4 μs. Any deviation from this standard set will be explicitly stated. We also note that as the electrolyte is rather dilute, correlations between the ions are assumed to be absent. With the increase of ion concentration, more effects have to be taken into account.^51^ All measurements are performed in the late-time limit-cycle regime, when all transient effects have vanished. This way, all time dependencies in the system have the same period as that of the driving voltage, with at most a phase difference as we will see.

Most of the previous work on AREF concentrated on asymmetric electrolytes containing ions with unequal diffusion coefficients driven by a harmonic (single-frequency sinusoidal) voltage.^38−44,52^ To appreciate the differences of AREF between these asymmetric electrolytes and the systems of interest here consisting of a symmetric 1:1 electrolyte (with equal diffusion coefficients) driven by the sawtooth potential of eq 6, we briefly recall the mechanism of AREF in the asymmetric case.

As was discussed in ref (47), the mechanism behind the creation of AREF in a system with an asymmetric electrolyte relies on the concentration *difference* of the faster (more mobile) ions gathering at the electrodes during a half-period *T*/2 and the slower (less mobile) oppositely charged ions during the complementary half period, an effect that is particularly strong for intermediate driving frequencies ωτ_RC_ ∼ 1. As a result, in the vicinity of both electrodes, the period-averaged concentration of the faster ions exceeds that of the slower ions, and the resulting period-averaged charge distribution *e*⟨*c*_+_ – *c*_–_⟩(*z*) in the electrolyte was found to be nonzero and results in a nontrivial period-averaged electrostatic potential ⟨Ψ⟩(*z*) and an associated period-averaged electric field ⟨*E*⟩(*z*) = −d⟨ψ⟩(*z*)/d*z*. Here, we defined the period average of a function *f*(*z*, *t*) as
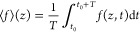
15where *t*_0_ is the (sufficiently late) time at which we start averaging. Because of the symmetry and equivalence between the two electrodes, at least at the period-averaged level, we find (for the asymmetric electrolyte with sinusoidal driving) perfect mirror symmetry with respect to the midplane for the period-averaged potential, so ⟨Ψ⟩(*z*) = ⟨Ψ⟩(−*z*), and likewise for the ionic concentrations and the charge density. The electric field, by contrast, exhibits perfect antimirror symmetry with respect to the midplane, thus ⟨*E*⟩(*z*) = −⟨*E*⟩(−*z*).^47^ As a consequence of this symmetry, it was found in ref (47) that a convenient integral quantity to characterize (the strength of) AREF was the time- and space-averaged (dimensionless) electric potential . An additional consequence of these (anti)symmetries combined with global charge neutrality was a vanishing period-averaged surface charge density ⟨σ⟩ on both electrodes at *z* = ± *L*/2, such that not only ⟨Ψ⟩(±*L*/2) = 0 but also ⟨*E*⟩(±*L*/2) = 0 for asymmetric electrolytes with symmetric driving voltages.

**Figure 3:**
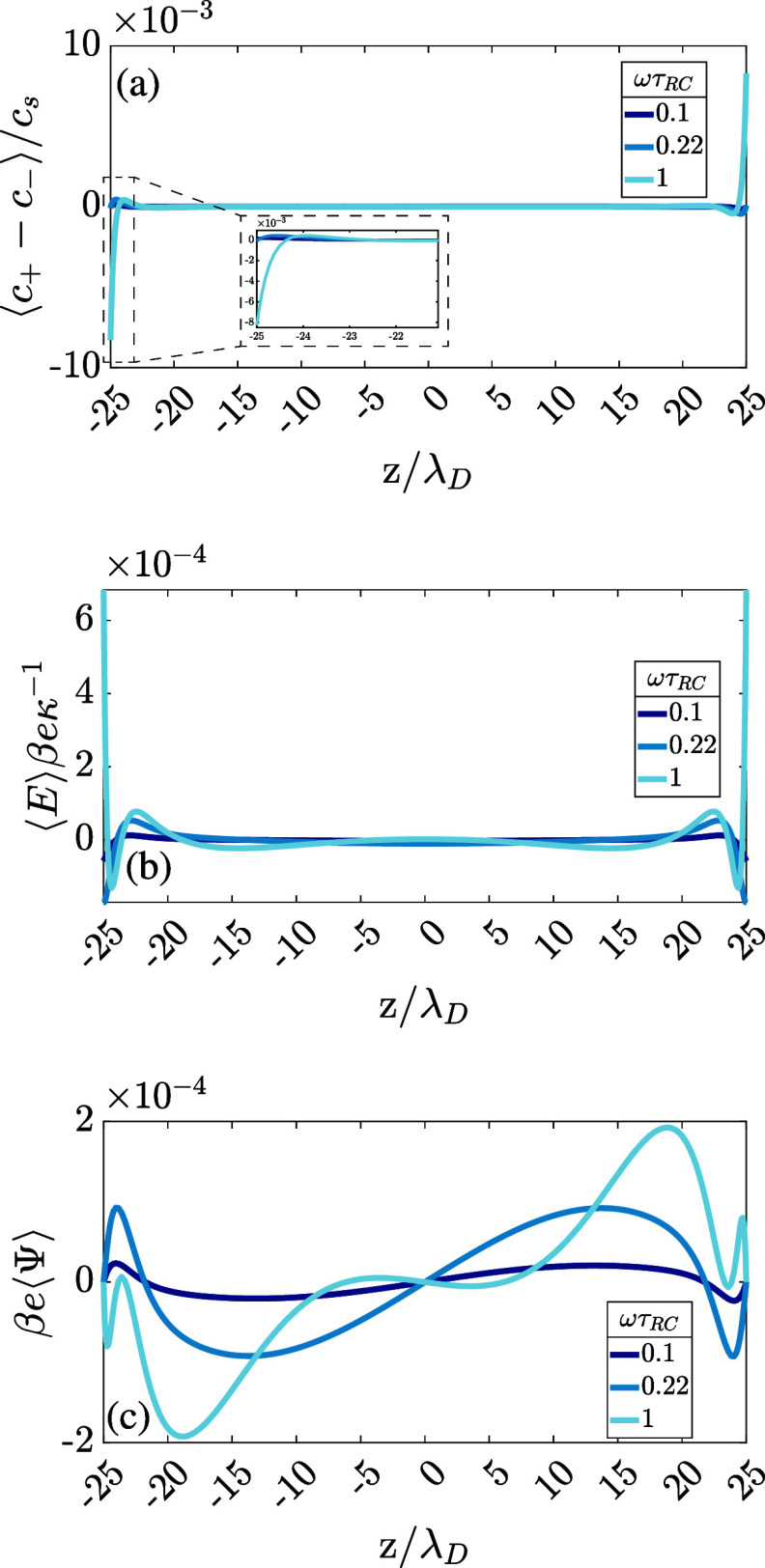
Time-averaged dimensionless spatial profiles of the (a) ionic charge density ⟨*c*_+_ – *c*_–_⟩/*c*_s_, (b) electric field β*e*κ^–1^⟨*E*⟩, and (c) electric potential β*e*⟨Ψ⟩ in a 1:1 aqueous electrolyte confined between two planar electrodes separated by distance *L* = 50λ_D_. The electrode at *z* = *L*/2 is grounded, whereas the one at *z* = −*L*/2 is driven by an AC sawtooth potential of eq 6 with amplitude Ψ_0_ = 3/β*e* = 75 mV. Three different driving frequencies ωτ_RC_ = 0.1, 0.22, and 1 with *RC* time τ_RC_ given by eq 13 are denoted with different colors.

Compared to the case of asymmetric ion diffusion coefficients that we just discussed, the system of a 1:1 electrolyte with equal ionic diffusion coefficients driven by the asymmetric sawtooth voltage has a different mechanism for AREF creation. This is immediately apparent from Figure 3a, that shows the numerical solution of the PNP equations of the period-averaged charge density profile ⟨*c*_+_ – *c*_–_⟩(*z*) for our standard parameter set. At the left electrode placed at *z* = −*L*/2, we see a period-averaged accumulation of negative ionic charge, whereas on the opposite side at *z* = *L*/2, an equal but opposite (positive) charge density accumulates in the vicinity of the electrode. Clearly, this charge density profile is antisymmetric with respect to mirroring in the midplane, ⟨*c*_+_ – *c*_–_⟩(*z*) = −⟨*c*_+_ – *c*_–_⟩(−*z*), which contrasts the mirror symmetry we encountered earlier in the cases of unequal ionic mobilities. Such an antisymmetric period-averaged charge distribution creates a perfectly mirror-symmetric AREF ⟨*E*(*z*)⟩, as also shown in Figure 3b, where we notice that the electric fields at *z* = ± *L*/2, so at the electrodes, do not vanish. This implies by the Gauss law that the period-averaged surface charge ⟨σ⟩ on the electrodes is nonzero in this case. At the same time, we see in Figure 3c that the period-averaged potential profile ⟨Ψ⟩(*z*) follows the antimirror symmetry of the charge distribution. As a consequence, its spatial average *U* will be identically zero, which implies that, unlike in ref (47), it cannot be used as a measure for the AREF strength. Instead, it is now natural to use the time-averaged surface charge density ⟨σ⟩ on the electrodes for this purpose, or rather its dimensionless version
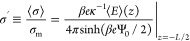
16where we introduced the Gouy–Chapman surface charge density σ_m_ = *e*(κ/λ_B_) sinh(β*e*Ψ_0_/2) ≈ 7.6 mC/m^2^ at the static voltage β*e*Ψ_0_ = 3 as a reference, with the Bjerrum and Debye length set to λ_B_ = *e*^2^/4πε_0_ε*k*_B_*T* ≃ 0.72 nm and κ^–1^ ≃ 10 nm, respectively.

**Figure 4:**
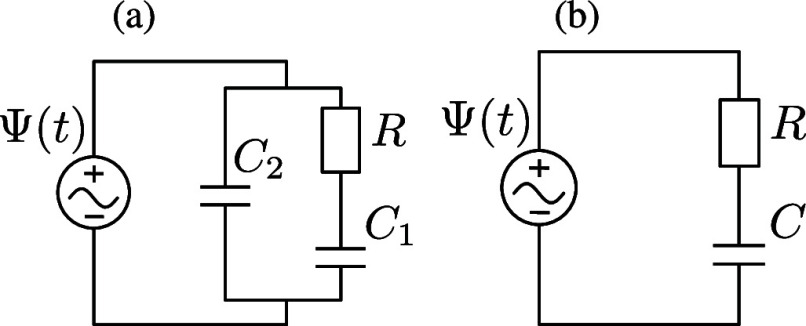
(a) Equivalent electric circuit corresponding to the large electrolytic cell with *L* ≫ κ^–1^ in the linear regime. Resistance and capacitance of the cell at infinite frequency are denoted by *R* and *C*_2_, respectively, whereas the total capacitance of two fully developed electric double layers at the electrodes is denoted by *C*_1_, as described by eqs 17 and 18. (b) Simplified equivalent electric circuit corresponding to the low-frequency case  with *C* = *C*_1_.

To understand the mechanism behind AREF in the present system, we will use the so-called equivalent circuit corresponding to the system that we are studying. It is well known that several aspects of electrolytic systems can often be approximated by equivalent electronic circuits,^53−57^ with ref (58) providing a historical overview on this matter. As was shown in ref (47), the system in Figure 2 can in the linear screening regime β*e*Ψ_0_ ≪ 1 be approximated by the circuit shown in Figure 4a, where the capacitors *C*_1_ and *C*_2_ and the resistor *R* take, for an electrolytic system of lateral area *A*, the form
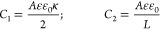
17
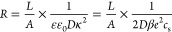
18Physically, *R* corresponds to the Ohmic resistance of the homogeneous aqueous electrolyte with monovalent charge carriers of concentration 2*c*_s_ and mobility β*D*, and *C*_1_ represents the capacity of the EDLs at the electrodes—it is the net capacity of the two fully developed EDLs in series, each with the linear-screening capacitance *A*εε_0_κ. Similarly, *C*_2_ represents the purely dielectric capacitance of a water-filled parallel-plate capacitor without any ionic charge carriers and characterized by the size *L* and area *A*.

**Figure 5:**
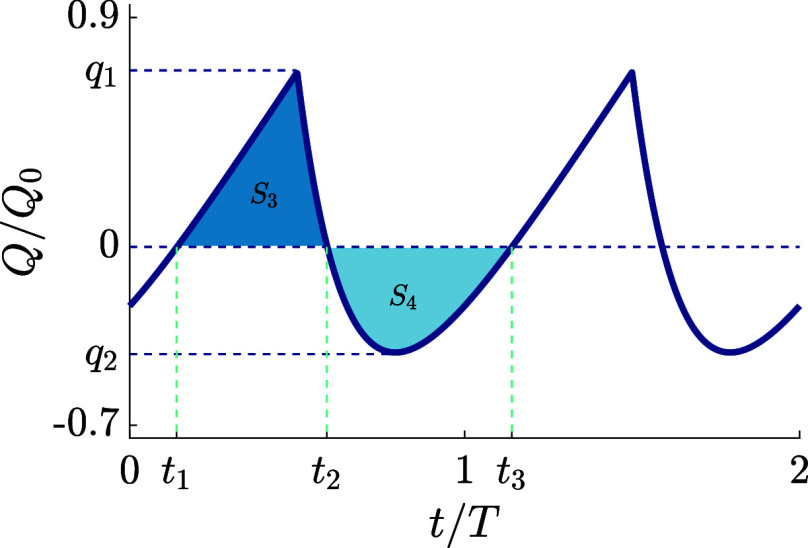
Time-dependent charge *Q*(*t*) (in units of *Q*_0_) as defined in eq 19 stored in the capacitor of the linear equivalent circuit of Figure 4b as a function of time for the full sawtooth potential Ψ(*t*) of eq 1. The asymmetry in the driving potential introduces not only an asymmetry of the positive and negative charge amplitudes, |*q*_1_| ≠ |*q*_2_|, but also of the time interval that the charge is positive or negative, *t*_2_ – *t*_1_ ≠ *t*_3_ – *t*_2_. For linear circuits, or linear screening, this translates into a vanishing period-averaged charge since *S*_3_ = *S*_4_ identically. In the nonlinear case of the electrolytic cell at high voltages, however, this condition gets violated and results in a nonzero period-averaged surface charge σ′ on the electrodes.

Despite the circuit of Figure 4a being only a quantitative mapping in the case of the linearized PNP equations valid at small driving potentials, it was demonstrated in ref (47) that a lot of qualitative information can still be extracted even in the nonlinear regime of interest here. At the same time, ref (47) also showed that for low frequencies ωτ_s_ ≲ 1, where , the circuit of Figure 4a can be successfully approximated by a simplified circuit shown in Figure 4b, which will be employed in this paper. Following the derivations in ref (47), and setting *C* = *C*_1_, we first analytically calculate the charge *Q*(*t*) accumulated in the capacitors of the circuit when the sawtooth driving voltage of eq 1 is applied, yielding
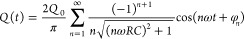
19where *Q*_0_ = Ψ_0_*C* is a reference charge and φ_n_ = arctan(1/(*n*ω*RC*)) is the *n*-th phase angle. In Figure 5, we plot two periods of *Q*(*t*)/*Q*_0_ as a function of (dimensionless) time *t*/*T* for the same driving as in Figure 1a (so for all harmonic modes rather than only two) for driving frequency ωτ_RC_ = 1. The phase shift between voltage and charge is evident. The plot identifies the two (dimensionless) times *t*_1_ and *t*_2_ in between which *Q*(*t*) > 0, and likewise the interval between *t*_2_ and *t*_3_ = *t*_1_ + 1 during which *Q*(*t*) < 0. The plot also shows the maximum *q*_1_, the minimum *q*_2_, and the integrated (absolute) surface areas *S*_3_ and *S*_4_ under the curve of *Q*(*t*)/*Q*_0_. We see for the present example that while the curve corresponding to the area *S*_3_ has a higher amplitude than that of the area *S*_4_, so |*q*_1_| > |*q*_2_|, the base of *S*_4_ is actually wider, Δ*t*_1_ ≡ *t*_2_ – *t*_1_ < Δ*t*_2_ ≡ *t*_3_ – *t*_2_. In the linear response regime, this is such that *S*_3_ = *S*_4_ when *S*_1_ = *S*_2_ in Figure 1a, which implies a vanishing period-averaged charge on the capacitor in this linearized case.

However, as we will see in more detail in the “Results and Discussion” section below, the electrolytic system of interest is in the nonlinear screening regime with a nonzero period-averaged (dimensionless) surface charge on the left electrode σ^′^ ∼ Ψ_0_^3^. This is a consequence of a nontrivial rescaling of the time-dependent electrode charge σ(*t*), that causes the analogues of the extrema *q*_1_ and *q*_2_ of the charge curve to scale nonlinearly with the voltage amplitude. In turn, this causes a nontrivial relation between the amplitude difference Δ*q* ≡ |*q*_1_| – |*q*_2_| and the base width difference Δ*t* ≡ |Δ*t*_1_ – Δ*t*_2_|, leading to a nonzero time-averaged area Δ*S* = *S*_3_ – *S*_4_ ≠ 0 and consequently to a nonzero time-averaged surface charge σ′ with a sign that depends on the system parameters, as we will see in the “Results and Discussion” section below.

## 2 Results and Discussion

**Figure 6:**
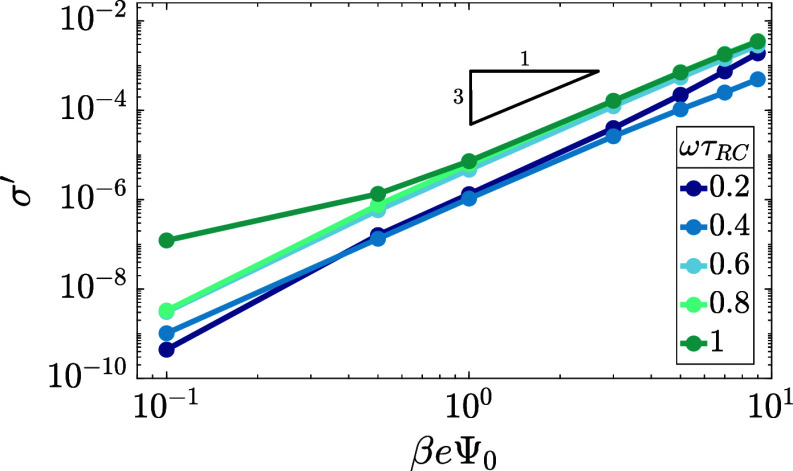
Period-averaged dimensionless surface charge σ′ of eq 16 plotted in the double-logarithmic representation against the driving voltage amplitude for varying driving frequencies ω at our standard parameter set (see the text). The cubic scaling σ^′^ ∼ Ψ_0_^3^ demonstrates that AREF is a nonlinear effect.

**Figure 7:**
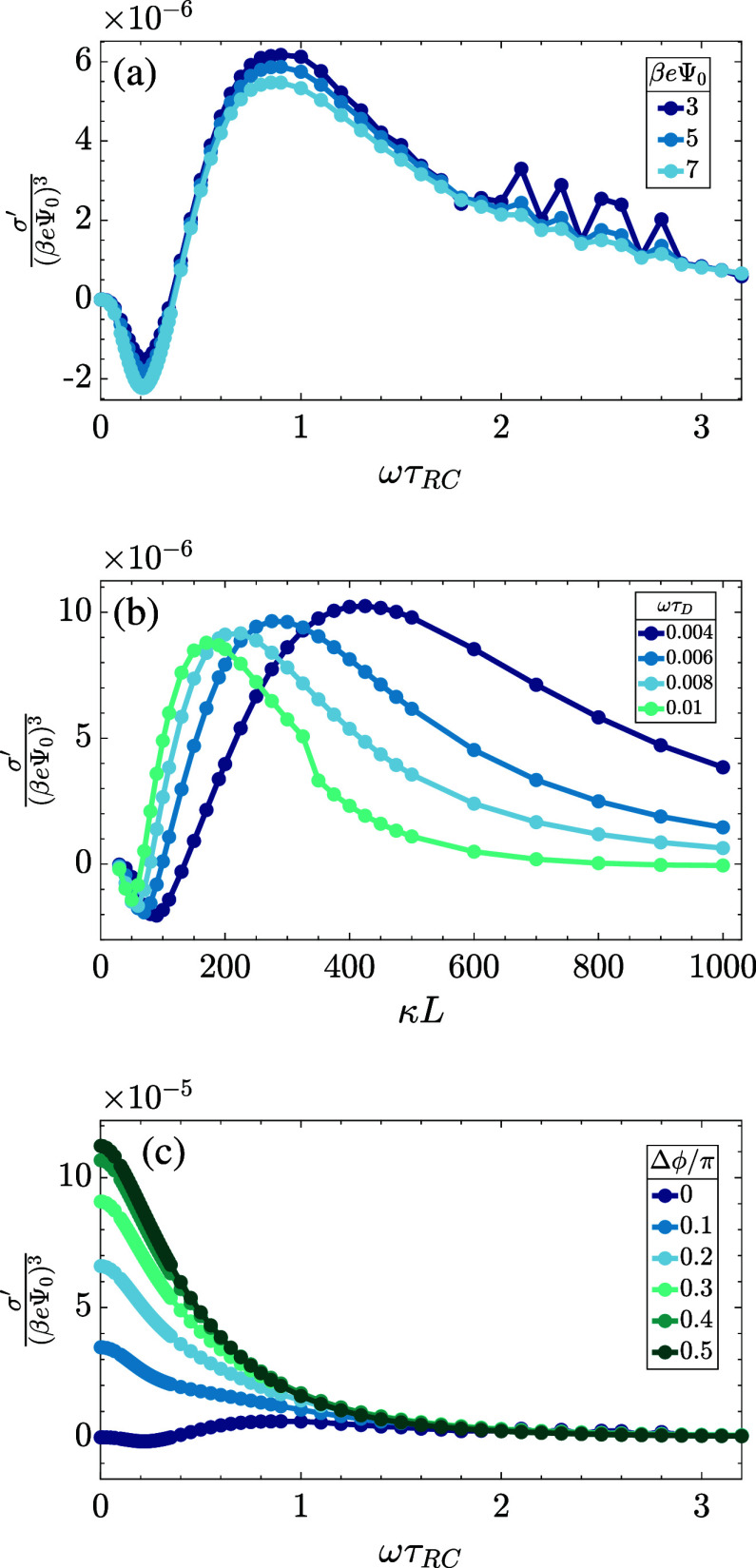
Numerically obtained period-averaged dimensionless surface charge  from late-time solutions of the PNP equations for the standard parameter set (see the text) plotted against the (a) dimensionless frequency ωτ_RC_ for several voltage amplitudes, (b) dimensionless system size κ*L* for several dimensionless driving frequencies ωτ_D_, and (c) driving frequency ωτ_RC_ for several phase shifts Δϕ of eq 20. In (a), we see a collapse of the curves for several voltage amplitudes Ψ_0_.

In this section, we study the dependence of the numerically obtained time-averaged surface charge σ′, defined in eq 16, on the main system parameters. We recall that all numerical calculations are performed using the two-term truncation of eq 2. The key results are presented in Figure 6, where we show that σ^′^ ∝ Ψ_0_^3^, and in Figure 7, where we plot , in (a) and (c) as a function of the driving frequency for different driving amplitudes (a) and different phase angles Δϕ between the two sinusoidal terms of the two-term sawtooth function in eq 2 (c) as we will see in more detail below, and in (b) as a function of the system size at several driving frequencies. In all cases shown in Figure 7, we see variations over an order of magnitude and even changes of the sign, which testify for the substantial tunability of AREF. However, we also see in Figure 6 that the order of magnitude of σ′ is at most of the order of 10^–3^, such that the period-averaged surface charge ⟨σ⟩ is at least 3 orders of magnitude smaller than the typical static Gouy–Chapman surface charge density σ_m_ at Ψ_0_ = 75 mV as defined below eq 16 for our system parameters. This does not imply, however, that AREFs are a mere quantitative effect without qualitative consequences, since the force that is exerted by an AREF on a (colloidal) body also depends on its net charge (which should therefore be large enough for AREF to be physically relevant, we estimate typically 3 orders of magnitude larger than the unit charge for the present (typical) parameters). Therefore, we will investigate, discuss, and interpret the dependence of AREF on the system parameters in more detail below.

### 2.1 Applied Voltage Amplitude

Similar to ref (47), the range that we consider for the driving voltage amplitude Ψ_0_ is limited from above by the point ion approximation, which even for *c*_s_ = 1 mM can give rise to unrealistically high local concentrations within the point-ion limit due to strong ion crowding effects that take place in actual electrolytes at the electrodes.^2,59,60^ This occurs beyond β*e*Ψ_0_ ≈ 8–9, which is therefore the upper limit that we consider in Figure 6, where we plot, for various driving frequencies, the dependence of σ′ on Ψ_0_ for our standard parameter set. The slope of the double-logarithmic curves is essentially identical to 3 across the range of frequencies ωτ_RC_ ∈ [0.2, 1] that we consider here, i.e., σ^′^ ∝ Ψ_0_^3^. This nonlinear scaling confirms that AREF is a nonlinear screening effect in the present case of a symmetric electrolyte driven by the sawtooth voltage, very similar to the earlier case of a sinusoidal voltage driving an asymmetric electrolyte as studied in refs (40, 42, and 47). This entices the further study of its dependence on frequency, the phase shift between the two harmonic modes of the driving voltage, and the system size in terms of the scaled form  below.

### 2.2 Frequency

In Figure 7a, we plot  as a function of the dimensionless frequency ωτ_RC_ for our standard parameter set at a number of voltage amplitudes Ψ_0_. As expected, the curves essentially collapse for all Ψ_0_ and decay to zero in the high- and low-frequency limits. We assign the irregularities in the graph for the lowest voltage in the high-frequency regime ωτ_RC_ ∼ 2–3 as numerical artifacts without any significant physical meaning, stemming from the small numbers involved. Interestingly, however, in the frequency range ωτ_RC_ ∼ 0.1–2 where the graphs are smooth, the average surface charge curves exhibit a change of sign while featuring both positive maximum at ωτ_RC_ ∼ 1 and a negative minimum at ωτ_RC_ ∼ 0.3. The mechanism that generates such curves can be best understood in the context of an “area competition” between *S*_3_ and *S*_4_ under the *Q*(*t*) curve for the equivalent circuit in Figure 5, as we discussed above, but now with the time-dependent surface charge density σ(*t*) obtained from the nonlinear PNP equations being the analogue of the capacitor charge *Q*(*t*) in the linear circuit.

**Figure 8:**
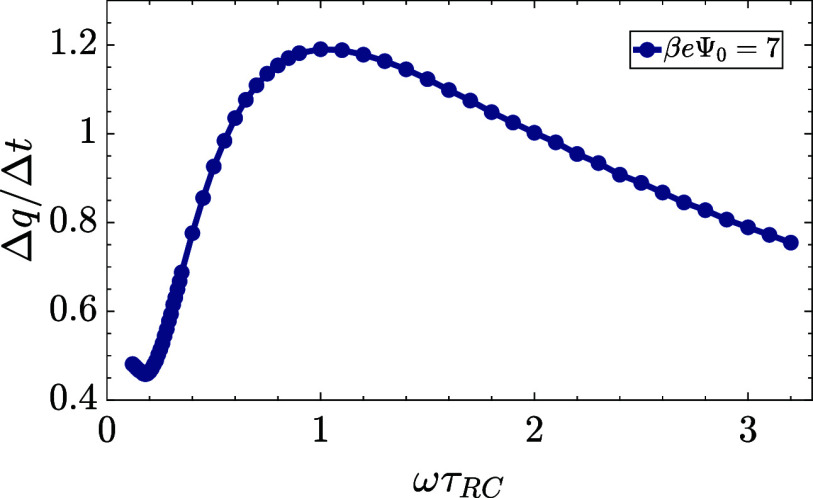
Ratio of the amplitude difference and time difference Δ*q*/Δ*t* for the numerical solution σ′ as a function of dimensionless frequency ωτ_RC_. Plotted for the standard parameter set (see the text), however, with β*e*Ψ_0_ = 7 to minimize the numerical noise seen at higher frequencies in Figure 7a. The shape of the Δ*q*/Δ*t*(ω) curve is remarkably similar to that of the σ′(ω) curve in Figure 7a.

Depending on the parameter range, the σ(*t*) analogue of either Δ*q* or Δ*t* dominates during a period of the (late time) voltage and charge oscillation, determining the sign of the time-averaged charge. To check this statement, we calculate (the analogues of) Δ*q* and Δ*t* for the numerical results of σ(*t*) (driven by the two-term sawtooth function) and plot their ratio Δ*q*/Δ*t* as a function of the dimensionless frequency ωτ_RC_ in Figure 8. Interestingly, comparing this ratio to the σ′(ω) curve in Figure 7a, we see a remarkable similarity in the shape of the curves, which suggests that a nontrivial competition between the amplitudes of the time-dependent surface charge and the duration of the time interval of its positive and negative sign is indeed able to explain the nontrivial nonmonotonic shape of the σ′(ω) curve of Figure 7a.

### 2.3 System Size

Figure 7b shows the dependence of  on system size *L* (in units of the Debye length) for various driving frequencies ωτ_D_ for our standard parameter set. Rather than using the dimensionless combination ωτ_RC_ of eq 13 to characterize the frequency of the driving voltage, here, we use ωτ_D_ defined in eq 14 as this combination does not depend on *L*. The maximum σ′ for the relatively large system sizes of interest, say in the range of κ*L* ∈ [10, 10^3^], occurs at larger κ*L* for lower frequencies ωτ_D_, and one checks that they all correspond to the regime where ωτ_RC_ ∼ 1. This agrees with our findings of Figure 7a. In fact, the dependence of σ′ on frequency in Figure 7a and on *L* in Figure 7b is very similar, which in retrospect is not surprising since the key dimensionless parameter ωτ_RC_ is linear in both *L* and ω.

### 2.4 Phase Shift

**Figure 9:**
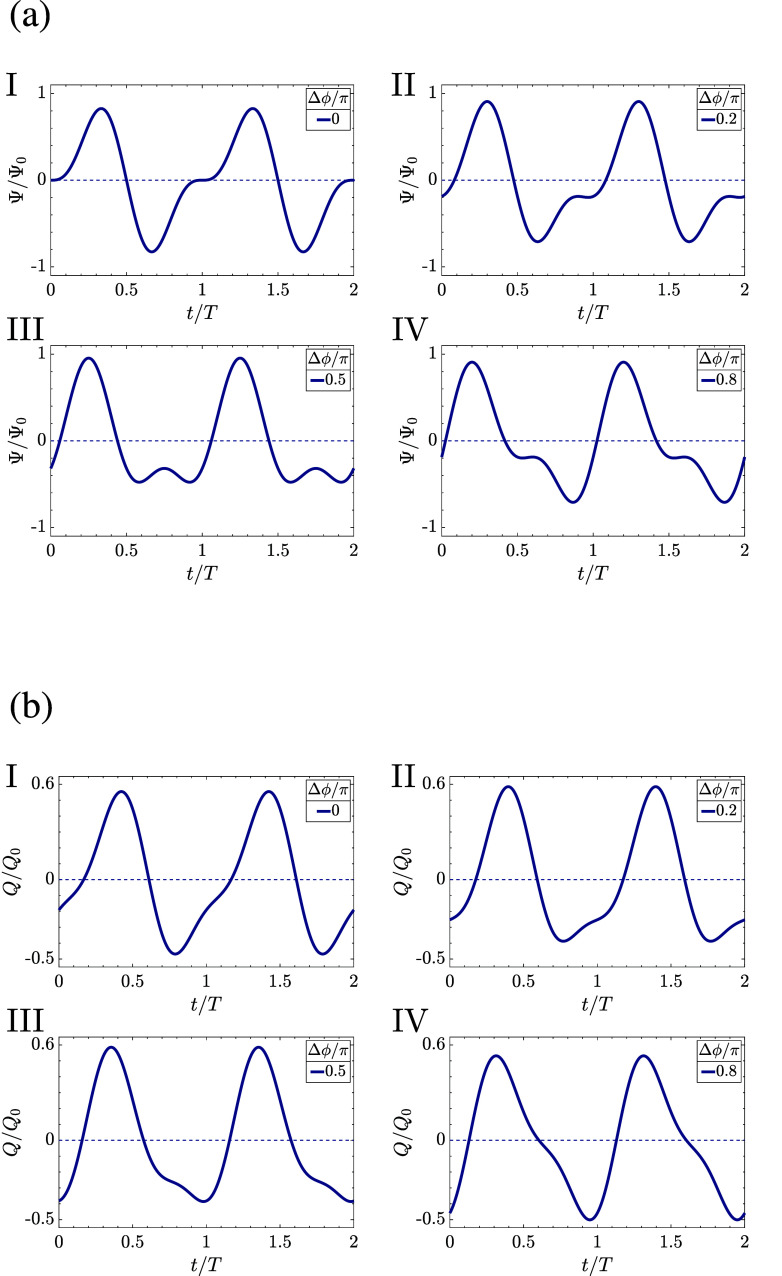
(a) Two-term sawtooth voltage of eq 20 for phase shifts Δϕ/π equal to (I) 0, (II) 0.2, (III) 0.5, and (IV) 0.8 and (b) resulting charges accumulating in the capacitors of the equivalent circuit of Figure 4b.

As was mentioned in the introduction, the main advantage of using a sawtooth function to drive a symmetric electrolyte in the system of Figure 2 compared to driving an asymmetric electrolyte with a sinusoidal voltage like in ref (47) is that one can manipulate AREF by simply altering the sawtooth potential without having to change the electrolyte properties (which would require the electrolyte to be changed in different experiments). As we are using the two-term sawtooth voltage of eq 6, it is thus interesting to see whether the AREF can be amplified or suppressed by shifting the relative phase Δϕ between two sinusoidal terms away from zero. For this reason, we consider the modified driving potential
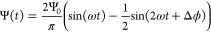
20which is identical to eq 2 for the case Δϕ = 0. We note that a nonzero phase shift keeps the period-averaged driving potential equal to zero while it does affect the rate of voltage change substantially and the maximum/minimum voltage during a period somewhat. We plot this driving potential in Figure 9a at phase shifts Δϕ/π = 0, 0.2, 0.5, and 0.8 in the panels I through IV, respectively, together with the charge *Q*(*t*) accumulated in the capacitor of the equivalent circuit of Figures 4b in 9b. As we see in Figure 9a, any of the three nonzero phase shifts increases the maximum and decreases the minimum of the driving voltage, resulting in an increase of Δ*q* in the corresponding plots of *Q*(*t*) in Figure 9b. At the same time, while Δ*t* changes with Δϕ, it does not get affected by the nonlinearity of AREF, thus it does not influence the surface charge dependence on the phase shift σ′(Δϕ). On this basis, one could expect a strong effect of Δϕ on the average surface charge σ′ in the nonlinear electrolytic cell.

**Figure 10:**
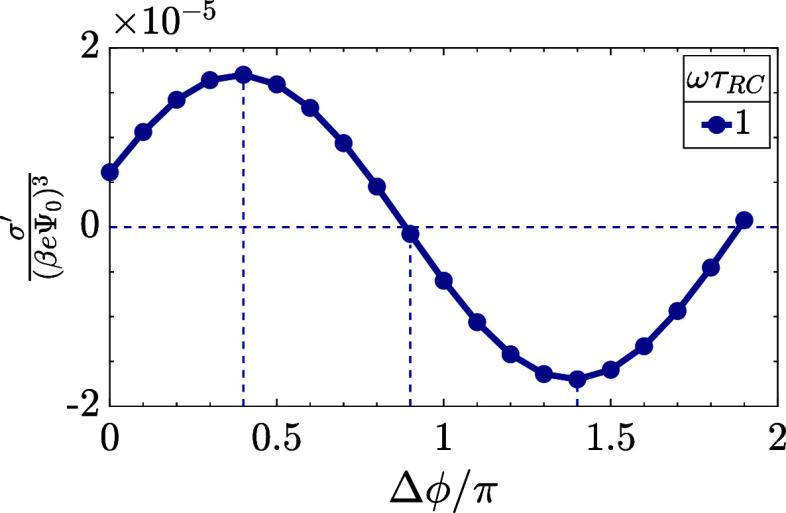
Dimensionless and scaled period-averaged surface charge  as obtained from numerical late-time solutions of the PNP equations for the standard parameter set (see the text) as a function of the phase difference Δϕ between the two sinusoidal terms of the two-term sawtooth potential of eq 2 at ωτ_RC_ = 1. For these parameters, the period-averaged surface charge has a maximum at Δϕ = 0.4π and a minimum (of opposite sign) at Δϕ = 1.4π.

This strong effect of the phase shift is indeed confirmed by Figure 7c, where we plot  as a function of the dimensionless frequency ωτ_RC_ for our standard parameter set at Δϕ/π = 0, 0.2, 0.5, and 0.8. We see that as we shift the phase, the AREF effect can actually increase by as much as an order of magnitude, reaching its highest values at Δϕ = 0.5π. At the same time, we see that it only changes sign with frequency for the case Δϕ = 0. Increase of Δ*q* with the phase shift is well reflected in Figure 10, where we plot σ′ as a function of the phase shift Δϕ at a fixed frequency of ωτ_RC_ = 1. As we see, the average surface charge has a maximum at Δϕ = 0.4π and a minimum at Δϕ = 1.4π, where it also has the opposite sign.

### 2.5 Sawtooth AREF vs Symmetric AREF

Here, we briefly compare the spatial dependence and the magnitude of AREF in the present case of a symmetric electrolyte with equal ionic diffusion coefficients driven by a sawtooth voltage with the more conventional case of an asymmetric electrolyte (with different ionic diffusion coefficients) driven by a sinusoidal voltage. We focus on the period-averaged electric field profile shown in Figure 3b for the present standard parameter set and the equivalent plot shown in Figure 2b of ref (47) for identical system parameters (β*e*ψ_0_ = 3, ωτ_RC_ = 1, κ*L* = 50) at a ratio of ionic diffusion coefficients equal to 2 and 3.5. A striking difference, discussed briefly before, concerns the differences in mirror symmetry with respect to the midplane. Also, for the case of sawtooth driving, we see two significant AREF peaks (a minimum and a maximum) of the same order of magnitude in the Debye-length vicinity of the electrodes, whereas in the case of the asymmetric electrolyte, we only obtain a single significant peak (a minimum at one electrode and a maximum at the other in agreement with the mirror antisymmetry). We also note that the scale of the AREF peaks is roughly an order of magnitude larger in the asymmetric case compared to the sawtooth case; however, the latter spreads almost twice as deep into the bulk of the electrolyte. We can assess the magnitude of the force exerted by such an AREF on a typical charged colloid immersed in the electrolyte. Considering a typical colloid of radius *a* = 150 nm at zeta potential ζ ≃ 75 mV in an aqueous 1:1 electrolyte of Debye length κ^–1^ = 10 nm, one estimates on the basis of the Gouy–Chapman relation a colloidal valency *Z* ∼ 10^4^.^61^ Estimating the resulting period-averaged net electric force acting on this colloid due to AREF as *Ze*⟨*E*⟩, we find for our standard parameter set on the basis of the results of Figure 3b a force of order β^–1^/κ^–1^, which is significant compared to the thermal forces since this implies that *Z*β*e*κ^–1^⟨*E*⟩∼ 1.

## 3 Conclusions

In this work, we investigate the time-averaged static electric field generated within the electrolytic cell depicted in Figure 2 when exposed to a sawtooth-shaped AC potential, under the condition of equal diffusion coefficients for monovalent cations and anions, i.e., *D*_+_ = *D*_–_. We numerically solve the coupled nonlinear PNP equations for ionic diffusion and migration in the cell to examine the dependence of the magnitude of the emerging AREF on key system parameters. These parameters include the amplitude Ψ_0_ of the applied AC sawtooth voltage, the driving frequency ω, the phase shift Δϕ between the lowest two harmonic modes of the driving potential, and the system size *L*, where we note that these system parameters can all be externally tuned without requiring a change of the electrolyte.

The asymmetry in the rate of change of the driving sawtooth voltage induces, despite the equal diffusion coefficients of the cations and anions and despite a zero period-averaged applied voltage, a nonzero period-averaged electrode charge ⟨σ⟩ that is responsible for a nonzero period-averaged AREF between the electrodes. While AREF fundamentally represents a nonlinear screening phenomenon that we find to be proportional to Ψ_0_^3^, we could still obtain additional insights by conducting an analysis using the linear RC circuit of Figure 4b that was also used and derived in ref (47). The analytic expression for the time-dependent charge *Q*(*t*) on the capacitor of this circuit, in particular, the difference between (i) the maximum and the minimum of this charge (represented by Δ*q*) and (ii) the duration of the time-interval of positive and negative charge (represented by Δ*t*), provides a clue on the physics of the nonlinear phenomenon of AREF. These nonzero differences have opposite effects on the period-averaged charge, which cancels identically even for nonzero Δ*q* and Δ*t* in the case of linear circuits. However, this cancellation is no longer exact in the nonlinear case of the PNP equations, where an intricate competition between Δ*q* (favoring a net positive charge for our parameter choices) and Δ*t* (favoring a net negative charge) depends sensitively on the system parameters. For driving frequencies ω that are of the same order as the inverse of the characteristic RC time of electric double layers, i.e., when ωτ_RC_ ∼ 1, this competition between Δ*q* and Δ*t* induces the most prominent period-averaged distribution of ionic charges, which, consequently, results in the largest nonzero AREF structure. The dependence on the system size *L* is largely reflected by the dependence on the RC time, which also depends on *L*. A relatively strong AREF effect of an order of magnitude can be induced by a phase difference Δϕ = π/2 between the two modes of the driving voltage in the two-mode approximation.

Finally, we noted that a recent investigation on floating colloids subjected to AC voltage within an electrolytic cell^41^ proposed that apart from AREF, DEP might also play a role in counteracting the gravitational forces on the colloids, depending on the system parameters. However, the relative contribution of each of these mechanisms to the floating height of the colloids remains an open question. It may well be possible to separate the contributions of the two mechanisms by employing sawtooth potentials, which we have shown here offer substantial opportunities for tuning AREF without the need to change the electrolyte or the colloidal suspension. We hope that this work stimulates experimental work along these lines to manipulate a given electrolyte externally.
